# Global Perspectives on Returning Genetic Research Results in Parkinson Disease

**DOI:** 10.1212/NXG.0000000000200213

**Published:** 2024-12-05

**Authors:** Ai Huey Tan, Paula Saffie-Awad, Artur F. Schumacher Schuh, Shen-Yang Lim, Harutyun Madoev, Azlina Ahmad-Annuar, J. Solle, Claire E. Wegel, Maria Leila Doquenia, Sumit Dey, Maria Teresa Perinan, Mary B. Makarious, Brian Fiske, Huw R. Morris, Alastair J. Noyce, Roy N. Alcalay, Kishore Raj Kumar, Christine Klein, for the Global Parkinson's Genetic Program (GP2), Yasser Mecheri

**Affiliations:** From the Division of Neurology (A.H.T., S.-Y.L.), Department of Medicine, Faculty of Medicine, University of Malaya, Kuala Lumpur, Malaysia; Programa de Pós-Graduação em Ciências Médicas da Universidade Federal do Rio Grande do Sul (P.S.-A.), Clínica Santa María, Santiago, Chile; Departamento de Farmacologia (A.F.S.S.), Universidade Federal do Rio Grande do Sul; Serviço de Neurologia (A.F.S.S.), Hospital de Clínicas de Porto Alegre, Brazil; Institute of Neurogenetics (H.M., M.L.D., C.K.), University of Lübeck, Germany; Department of Biomedical Science (A.A.-A.), Faculty of Medicine, University of Malaya, Kuala Lumpur, Malaysia; The Michael J. Fox Foundation for Parkinson's Research (J.S., B.F.), New York; Department of Medical and Molecular Genetics (C.E.W.), Indiana University, Indianapolis; Department of Neuroscience and Brain Health (M.L.D.), Metropolitan Medical Center, Manila, Philippines; Centre for Preventive Neurology (S.D., M.T.P., A.J.N.), Wolfson Institute of Population Health, Queen Mary University of London, United Kingdom; Unidad de Trastornos del Movimiento (M.T.P.), Servicio de Neurología y Neurofisiología Clínica, Instituto de Biomedicina de Sevilla, Hospital Universitario Virgen del Rocío/CSIC/Universidad de Sevilla, Spain; Laboratory of Neurogenetics (M.B.M.), National Institute on Aging, National Institutes of Health, Bethesda, MD; Department of Clinical and Movement Neurosciences (M.B.M., H.R.M.), UCL Queen Square Institute of Neurology, University College London, United Kingdom; Department of Neurology (R.N.A.), Columbia University Irving Medical Center, New York; Movement Disorders Division (R.N.A.), Neurological Institute, Tel Aviv Sourasky Medical Center and Tel Aviv School of Medicine, Tel Aviv University, Israel; Molecular Medicine Laboratory and Neurology Department (K.R.K.), Concord Clinical School, Concord Repatriation General Hospital, The University of Sydney; Translational Neurogenomics Group (K.R.K.), Genomic and Inherited Disease Program, Garvan Institute of Medical Research; and St Vincent's Healthcare Campus (K.R.K.), Faculty of Medicine, UNSW Sydney, Darlinghurst, New South Wales, Australia.

## Abstract

**Background and Objectives:**

In the era of precision medicine, genetic test results have become increasingly relevant in the care of patients with Parkinson disease (PD). While large research consortia are performing widespread research genetic testing to accelerate discoveries, debate continues about whether, and to what extent, the results should be returned to patients. Ethically, it is imperative to keep participants informed, especially when findings are potentially actionable. However, research testing may not hold the same standards required from clinical diagnostic laboratories and hold significant psychosocial implications. The absence of universally recognized protocols complicates the establishment of appropriate guidelines.

**Methods:**

Aiming to develop recommendations on return of research results (RoR) practice within the Global Parkinson's Genetics Program (GP2), we conducted a global survey to gain insight on GP2 members' perceptions, practice, readiness, and needs surrounding RoR.

**Results:**

GP2 members (n = 191), representing 147 institutions and 60 countries across 6 continents, completed the survey. Access to clinical genetic testing services was significantly higher in high-income countries compared with low- and middle-income countries (96.6% vs 58.4%), where funding was predominantly covered by patients themselves. While 92.7% of the respondents agreed that genetic research results should be returned, levels of agreement were higher for clinically relevant results relating to pathogenic or likely pathogenic variants in genes known to cause PD or other neurodegenerative diseases. Less than 10% offered separate clinically accredited genetic testing before returning genetic research results. A total of 48.7% reported having a specific statement on RoR policy in their ethics consent form, while 53.9% collected data on participants' preferences on RoR prospectively. 24.1% had formal genetic counselling training. Notably, the comfort level in returning incidental genetic findings or returning results to unaffected individuals remains low.

**Discussion:**

Given the differences in resources and training for RoR, as well as ethical and regulatory considerations, tailored approaches are required to ensure equitable access to RoR. Several identified strategies to enhance RoR practices include improving informed consent processes, increasing capacity for genetic counselling including providing counselling toolkits for common genetic variants, broadening access to sustainable clinically accredited testing, building logistical infrastructure for RoR processes, and continuing public and health care education efforts on the important role of genetics in PD.

## Introduction

Over the past 3 decades, there has been accumulating evidence supporting an important role for genetics in the development and progression of Parkinson disease (PD),^1-3^ and genetic testing in PD is becoming more commonplace across clinical, research, and direct-to-consumer settings.

The Global Parkinson's Genetics Program (GP2) is a major endeavor aiming to discover novel insights into the genetic drivers of PD and to make this knowledge globally available and actionable.^4^ This ambitious program aims to perform genotyping and/or sequencing in ∼200,000 individuals with PD and prioritizes the inclusion of populations worldwide that historically have been underrepresented in genetic studies.^5,6^ While the bulk of collected samples were initially planned to undergo genotyping using a high throughput and cost-effective custom-designed content platform (i.e., the NeuroBooster array),^7^ it is now anticipated that with the ongoing reduction in the costs associated with whole-genome sequencing, many samples will be sequenced, thus increasing the power to detect nearly all forms of genomic variation in an unbiased manner.^8-10^

Currently, the yield of genetic testing in PD in most settings is ∼5%–15%, depending on the population studied and the platform used (most commonly targeted gene panels or single-gene studies).^11-13^ However, this has been shown to be as high as 40%–50% in some populations.^3,14-17^ Known PD/parkinsonism genes have an autosomal dominant (e.g., *SNCA*, *LRRK2*, and *VPS35*), autosomal recessive (e.g., *PRKN*, *PINK1*, and *PARK7*/*DJ-1*) or X-linked (*TAF1*) mode of inheritance. In addition, risk genes are recognized, and in particular, carriers of *GBA1* variants have increased susceptibility to developing PD.^18^ New monogenic causes of PD continue to be discovered, such as *RAB32*, which was found in several populations in Africa, North America, and Europe.^19^ During the course of the GP2, it is expected that a large number of variants in PD genes with potential clinical relevance will be detected in a research setting.

The main purpose of a research program (such as the GP2) is to advance scientific understanding and gain mechanistic insights with the potential to benefit populations of people with PD.^20^ This is distinct from clinical testing, which is usually focused on attaining a diagnostic result which would then be used to inform clinical management.^20^ Traditionally, genetic results from research studies were not returned (for a variety of reasons discussed further below, including posing an “untenable burden on research infrastructure,” because disclosure can be resource-intensive)^20^; however, this practice is evolving. Practices for returning genetic research results also vary widely across different countries as a reflection of regional differences in the expertise and training of clinicians, the availability of genetic counselling resources, access to clinically accredited genetic testing, and attitudes of patients and the community.^21,22^ Some countries have adapted wide-scale research genetic testing such as the 100,000 Genomes Project in the United Kingdom, that later as the project developed, obtained diagnostic accreditation.^10^ In addition, there are important ethico-legal considerations, which include the participant's right of access to their personal data, the participant's right to know and right not to know, and the researchers' duty of care.^23^

The challenge of disclosing individual genetic findings to research participants presents both opportunities and risks, necessitating thoughtful consideration. Disclosing individual genetic research results can have direct benefits to participants, such as modifying medical management and providing more information regarding diagnosis and prognosis as well as opportunities for participation in clinical trials.^3,24,25^ Furthermore, there is a high level of interest and a general willingness of health professionals and researchers to return results, particularly if the results are thought to be clinically relevant and reliable.^8,24,26^ However, there are potential risks to returning research results, including the possibility of adverse psychological consequences to the participants and their family members—although some would argue that this has sometimes been overstated, and indeed positive implications on healthy behavior change have been reported.^27-29^ While GP2 strives for the highest quality of research results, the very stringent quality control measures that accredited diagnostic testing laboratories have to adhere to cannot usually be matched in the research setting, and errors such as mislabelling or mix-up of samples can occur.^30^ Moreover, despite the personal utility that individuals with PD derive from genetic test results, this area remains underexplored, especially in underrepresented and resource-constrained regions.^31^

Aiming to develop recommendations on return of results (RoR) practices within the GP2, we conducted a global survey to gain insights into the GP2 members' perceptions, practices, readiness, and needs on returning results of genetic research testing. Here, we seek to better understand the demand for RoR and the potential challenges and risks of RoR in a diverse range of countries and settings. The results of the survey may help with the design of suitable approaches to return genetic research results to patients with PD and families efficiently and safely, now and in the future.

## Methods

### Development and Execution of the GP2 Return of Results Survey

The survey was developed by 6 movement disorder neurologists with expertise in PD genetic testing from North and South America, Europe, Asia, and Australia, who are members of the GP2 RoR Interest Group (A.H.T., K.R.K., P.S.A., A.F.S.S., R.N.A., and C.K.). The contents of the initial survey draft were discussed in online meetings. Each item of the survey was refined through 2 rounds of appraisal for content validity, relevance, clarity, and conciseness. The draft was then converted into an online format, accessible through different browsers and devices. Readability and usability of the online survey were tested by the working group members and an additional movement disorder neurologist and a medical geneticist. During each step, items that were unclear were revised accordingly. The online survey consisted of 4 sections of multiple-choice questions: (1) demographics, (2) access to genetic testing services in clinical practice, (3) perceptions and ethical considerations on returning genetic research results, and (4) readiness to return genetic research results (eAppendix 1).

Invitation to participate in the online survey was sent via email to 572 GP2 members including 415 GP2 investigators and 157 GP2 trainees (e.g., postgraduate students or trainees in related clinical, genetic, and/or basic science GP2 projects) with 2 rounds of reminder emails. To improve the response rate, we addressed each GP2 member and explained the importance of the survey in developing a workflow for RoR in the GP2 in our invitation emails. Each GP2 member received an individualized survey link, which also enabled easy return to the survey at other times, until final submission. To ensure no missing survey data, each respondent was prompted to answer all the questions in one section before proceeding to the next section. A message of survey receipt and appreciation was sent on submission of the survey. Descriptive data and chi-square analyses were conducted using the IBM SPSS ver.23.

### Standard Protocol Approvals, Registrations, and Patient Consents

This study has obtained ethics approval from the University of Malaya Medical Centre Medical Research Ethics Committee (MREC ID NO: 2024625-13862). This online survey involved the collection of nonsensitive and anonymous data from GP2 investigators, a formal written consent form was not required by the ethics board. No patients or probands were involved in this survey.

## Results

**Figure 1:**
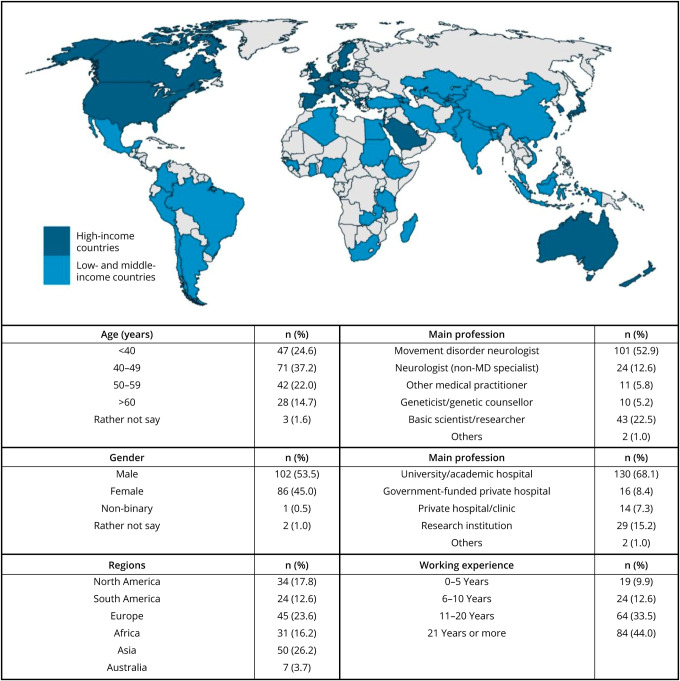
Demographics of 191 Survey Respondents Highlighted in blue in the map are 60 countries represented by the survey respondents; high-income countries are colored in dark blue, while low- and middle-income countries are colored in light blue. The table summarizes the age, sex, regions that the respondents originate from, main profession, main workplace, and years of working experience in the health care field of the surveyed cohort. MD = movement disorder.

A total of 191 GP2 members representing 147 institutions and 60 countries across 6 continents completed the online survey between July 27 and August 17, 2023. The survey response rate was 39.3% (n = 163/415) and 17.8% (n = 28/157) in the GP2 investigator and trainee groups, respectively. All submitted surveys had a 100% completion rate for each section. Respondent demographics are summarized in Figure 1. The highest numbers of respondents were from Asia (26.2%) and Europe (23.6%). Notably, 49.7% were from resource-limited regions (i.e., low- and middle-income countries [LMIC], as defined by the World Bank^22^). 71.3% were clinicians (101 movement disorder neurologists, 24 neurologists, and 11 other medical practitioners), while 22.5% were basic scientists/researchers and 5.2% were geneticists/genetic counsellors. More than two-thirds were working in university or academic teaching hospitals. Three-quarters (77.5%) of the respondents had >10 years of working experience in the health care field.

### Access to Genetic Testing in Clinical Practice

Among the 136 clinician respondents, 75% (n = 102/136) reported having access to genetic testing in clinical practice, through clinical diagnostic laboratories in their institutions/countries (n = 90/136) or outside their countries (n = 34/136), or through genetic research laboratories (n = 75/136). Table 1 presents the differences in access to genetic testing and counselling services between respondents from high-income countries (HIC) vs LMIC. Significantly larger proportions of respondents from HIC had access to genetic testing in clinical practice compared with those from LMIC (96.6% vs 58.4%, *p* < 0.001), where respondents from Africa and South America reported the lowest rates of access. Genetic testing in clinical practice was primarily paid for through government funding in HIC, while out-of-pocket payment was the primary funding mechanism for genetic testing in LMIC. Overall, 75.7% of clinician respondents (n = 103/136) reported having access to genetic counselling services, with higher service availability in HIC vs LMIC (88.1% vs 66.2%, *p* = 0.004). In LMIC, respondents reported higher access to genetic counselling services by neurologists or movement disorder neurologists (51.4%), compared with services by geneticists or genetic counsellors (35.7%). 25.4% of the respondents in HIC had access to genetic telemedicine services.

**Table 1:** Access to Genetic Testing and Counseling Services in Clinical Practice: Comparison Between High-Income vs Low- and Middle-Income Countries

Survey items	Response from all clinicians (n = 136)	Response from clinicians in HIC (n = 59)	Response from clinicians in LMIC (n = 77)	*p* Value
	% Answered yes	% Answered yes	% Answered yes	
Access to genetic testing in clinical practice				
Has access to genetic testing in clinical practice	75.0	96.6	58.4	<0.001^a^
Avenues for genetic testing in routine clinical practice				
Clinical diagnostic laboratory in own institution	38.2	67.8	15.6	<0.001^a^
Research genetic laboratory in own institution	41.2	71.2	18.2	<0.001^a^
Other clinical diagnostic laboratory in own country	52.9	69.5	40.3	0.001^a^
Other research genetic laboratory in own country	22.8	33.9	14.3	0.008^a^
Clinical diagnostic laboratory outside the country	25.0	28.8	22.1	0.426
Research genetic laboratory outside the country	21.3	25.4	18.2	0.399
Funding for genetic testing in routine clinical practice				
Government funding	39.7	61.0	23.4	<0.001^a^
Out-of-pocket funding (by patients)	58.1	40.7	71.4	<0.001^a^
Private insurance/prepaid funding	33.8	42.2	27.3	0.071
Development assistance funding	4.4	3.4	5.2	0.697
Access to genetic counselling services				
Has access to genetic counselling services	75.7	88.1	66.2	0.004^a^
Has access to genetic counselling by geneticist or genetic counsellor	54.0	77.8	35.7	<0.001^a^
Has access to genetic counselling by neurologist or MD neurologist	58.1	66.7	51.4	0.101
Has access to genetic telemedicine services	14.0	25.4	5.2	0.001^a^

Abbreviations: HIC = high-income countries; LMIC = low- and middle-income countries.

Comparisons between responses from HIC and LMIC were analyzed using χ-square; MD = movement disorder.

a Denotes statistical significance.

## Perceptions on and Current Practices in Returning Genetic Research Results

**Figure 2:**
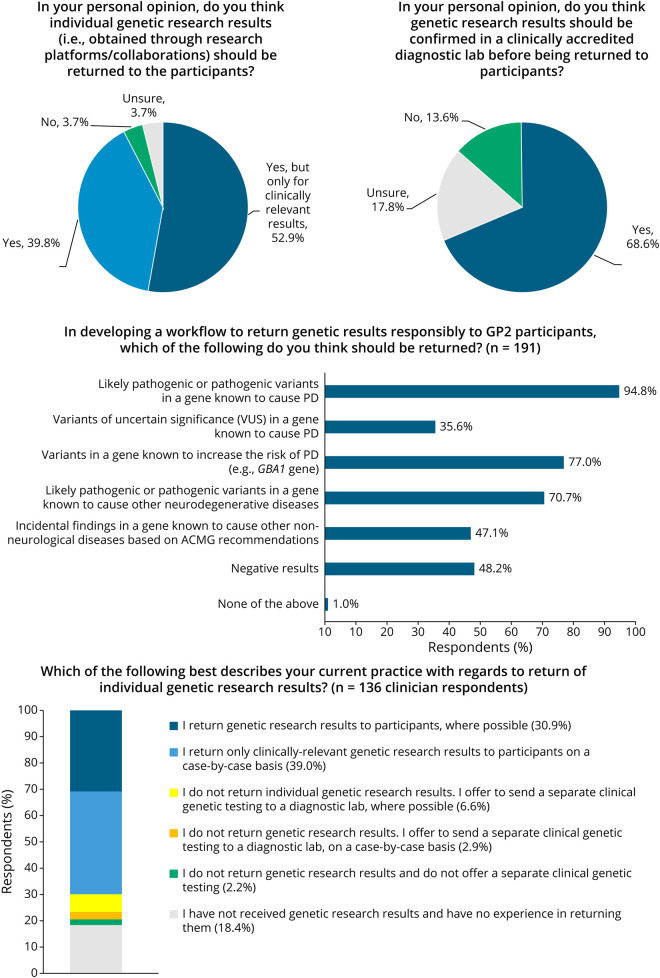
Perceptions and Current Practice on Return of Genetic Research Results

Figure 2 summarizes respondent perceptions on and current practices in returning genetic research results. Of the 191 respondents, 92.7% were of the opinion that individual genetic research results should be returned to research participants, although 52.9% felt that only clinically relevant results should be returned. 68.6% felt that genetic research results should be confirmed in a clinically accredited diagnostic laboratory before being returned to participants, while 17.8% were unsure. A substantial majority (70.7%–94.8%) felt that results regarding pathogenic or likely pathogenic variants in a gene known to cause PD or other neurodegenerative diseases, as well as variants known to increase the risk of PD (e.g., *GBA1* variants) should be returned. Slightly under half (47.1%–48.2%) responded that ACMG-recommended incidental findings and negative results should be returned.

Most of the clinician respondents (69.9%) practiced returning genetic research results directly to participants, while 9.5% offered separate clinical genetic testing through a diagnostic laboratory, and 2.2% did not return genetic research results or offer separate diagnostic testing. The top 5 major concerns in returning genetic research results included (1) a lack of resources to validate genetic research results, (2) potential errors in genetic research results, (3) a lack of informed consent from research participants on RoR, (4) lack of pretest genetic counselling during research recruitment, and (5) lack of experience/expertise in returning genetic results (eTable 1). Regarding potential implications on/issues surrounding research participants, the top 5 major concerns included (1) possible impact on family members, (2) psychological consequences (e.g., stress, anxiety, and depression), (3) low health literacy and basic understanding of genetics, (4) lack of access to new therapeutics or clinical trials, and (5) potential negative impact on insurance (eTable 1).

### Ethics and Local Regulations on Return of Results

Of 191 participants, 54 participants (28.2%) from 28 countries were aware of existing laws, policies, or guidelines governing or guiding RoR in their countries. Six participants from 3 countries stated that their local regulations do not allow RoR. 65 participants (34%) stated that there were no such local regulations, while the remaining 66 participants (34.6%) were unsure. There were instances of discordance between participants from the same country in their responses regarding the existence of local regulations, e.g., 9 of 29 participants from the United States considered that local regulations allowed RoR, 4 thought this was not permitted, 6 thought that there were no local regulations, and the remaining 10 were unsure.

A total of 93 participants from 73 institutions reported that their institutional ethics consent form contained a specific statement on RoR, whereby 37.0% could return genetic research results, 23.9% could return only clinically relevant results, 8.7% would obtain validation in a clinically accredited laboratory before returning the results, 16.3% would not return research results, 4.3% had other RoR approaches, while 9.8% were unsure regarding their ethics statements on RoR. Although there was also some discordance in the responses by participants from the same institution regarding their institutional ethics statement, these were more consistent compared with the responses on local regulations. About half (53.9%) of the 191 respondents collected responses from participants during recruitment on whether they would like their genetic results to be returned, while 64.9% felt that the majority (>50%) of their participants would like to know their genetic results.

## Readiness to Return Genetic Research Results

**Figure 3:**
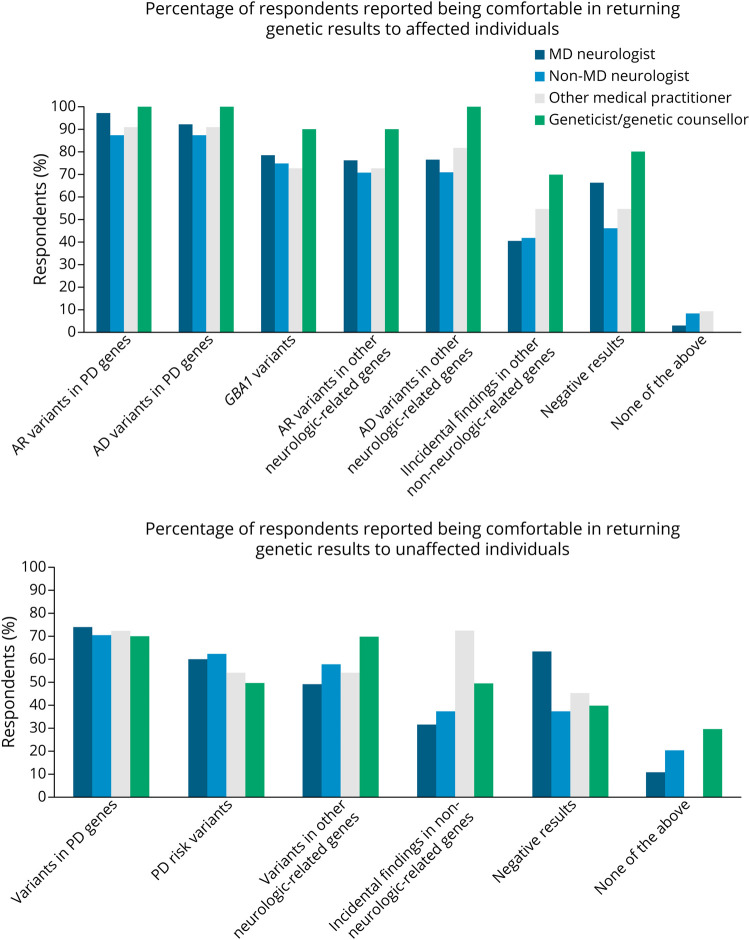
Comfort in Returning Genetic Research Results to Affected and Unaffected Individuals AD = Autosomal dominant; AR = Autosomal recessive; MD = movement disorder; PD = Parkinson disease

Overall, 46 of 191 respondents (24.1%) had formal training in genetic counselling, 31 of these respondents were clinicians. Among the 136 clinician respondents, the majority reported being comfortable in returning genetic research results (62.5% comfortable or very comfortable, 25% neutral, 9.6% slightly uncomfortable, and 2.9% not comfortable). Notably, the proportion of clinician respondents who were comfortable or very comfortable returning results was higher among those who had formal training in genetic counselling (87.1% vs 69.5%, *p* < 0.001). Comfort levels in returning genetic research results differed according to different types of genetic variants (Figure 3). Overall, for affected individuals, most (>85%) respondents were comfortable returning clinically relevant pathogenic variants in PD genes and >70% were comfortable returning the results on *GBA1* variants or pathogenic variants in other neurologic disorder-related genes. Of interest the level of comfort in returning negative results was lower (45.8%–80%) than returning positive results in genes associated with PD or related neurologic disorders (70.8%–100%). Only about 40% of movement disorder neurologists and non-MD neurologists were comfortable returning incidental findings to affected individuals. In general, a smaller proportion of respondents were comfortable returning results to unaffected individuals, about 70% were comfortable returning clinically relevant variants in PD genes, while about half were comfortable returning results on PD risk and causative variants in other neurologically related genes.

Of the 136 clinician respondents, 89% reported the ability to recollect new samples from their research participants for validation studies, and 49.6% estimated that they would be able to recollect samples from >50% of their cohort submitted to GP2. Among the respondents from HIC, 77.1% reported access to an accredited clinical diagnostic laboratory to validate selected GP2 results, while only 37.9% of respondents from LMIC reported similar access.

Of the 191 respondents, 80.1% indicated a desire to receive additional information or training on how to return genetic research results. Among the different training platforms, certified training programs were the most preferred, followed by in-person training workshops, on-demand online modules, live online training courses, and digital reading materials.

## Discussion

In this global survey of clinicians, researchers, and other professionals involved in PD genetics research, we uncovered novel insights and actionable findings in perceptions, practice, and readiness surrounding the return of genetic research results. It is important that an overwhelming majority (>90%) of respondents felt that individual genetic research results should be returned, consistent with previous studies conducted among stakeholders and patients in genomics research.^26,32^ The respondents divided on their view on the necessity of a clinical validation: two-thirds held the view that genetic research results should be validated in a clinically accredited diagnostic laboratory, but only a very small proportion (<10% of the respondents) offered separate clinically accredited genetic testing before RoR, likely reflecting current limitations in access to clinical genetic testing, and varying standards and practices around the world.^22^ We also identified important differences in access, resources, and training for genetic testing and validation, as well as ethical and regulatory considerations, between different institutions, countries, regions, and socioeconomic strata. Formal training in genetic counselling is lacking, and notably, the level of comfort in returning incidental genetic findings or returning results to unaffected individuals remains low.

A total of 52.9% of our survey respondents felt that only clinically relevant results (i.e., potentially diagnostic pathogenic/likely pathogenic variants in PD genes), rather than incidental or additional findings, should be returned. The potential effect on family members and psychological consequences were rated as top concerns. However, contrary to these common concerns, most participants from 2 large PD research cohorts in the United States reported no major adverse psychological effects from genetic result disclosure.^28,33^ In 2 separate studies, participants were prospectively offered choices regarding return of genomic results; 76.1%–94.5% chose to learn all genetic results including incidental findings, while 5.5%–14.4% chose a subset of results; only 0.5% of participants changed their choices after enrolment.^34,35^ While there may be hesitation in returning positive genetic results to unaffected individuals, in one survey, 46.1% of patients with PD indicated they would have liked to know about their risk for PD, even in the absence of disease-modifying therapy.^36^ Taken together, strategies for RoR should embrace the heterogeneity of participants' choices and personal preferences/values, as well as the evolving understanding on the impact of genetic results that may influence these choices (e.g., more defined knowledge on the natural history of the disease, penetrance, and treatment options). Dynamic forms of RoR consent allowing for changes in choices over time may be ideal,^34,37^ but will require more extensive allocation of resources to put into practice. It is important that all involved should bear in mind that the ethical principle of autonomy also gives participants the right *not* to know their genetic result, and unwanted research information should never be forced onto participants.^8,24,26^

Ethical considerations in genomics research involve striking a balance between the potential benefits of returning individual genetic results (such as informing medical management or contributing to a participant's understanding of their condition and assisting family and career planning, as well as enhancing research participation), and the risks (which include psychological harm, and the potential for misdiagnosis with lower quality control measures in research settings compared with clinical diagnostics). Legally, the situation is further complicated by country-specific requirements. For instance, in the United States, there are restrictions on the disclosure of results from laboratories that are not certified by the Clinical Laboratory Improvement Amendments (CLIA; through which the Centers for Medicare and Medicaid Services regulates human laboratory testing in the United States), highlighting the need for adherence to specific certification standards, which can be logistically impossible to be implemented within a global research program such as the GP2. The legal framework varies significantly across different countries, reflecting disparities in expertise, the availability of genetic counselling resources, and access to clinically accredited genetic testing. The lower access to genetic testing and related services in LMIC as highlighted in this survey and other previous reports^22,38,39^ may also influence local policies regarding RoR. For example, in some LMIC, the ethics committees may favor the disclosure of research results, even with their limitations, because this may be the only avenue available for testing to be done. In addition, there needs to be an awareness on the implications to other family members and future offspring including potential stigma that may be faced with genetic diagnosis in certain populations.^22^ Any approach to RoR must navigate this complex ethicolegal landscape and support tailored strategies that respect local regulations and cultural sensitivities while striving to uphold the highest ethical and scientific standards in genetics research.

While there is now broader acceptance that there are many ethical and pragmatic reasons to return clinically actionable genetic results, the practice of RoR raises several practical issues. While the majority of GP2 researchers expressed a willingness to return results, we found that only a quarter possessed formal genetic counselling training. Not surprisingly, a significant proportion found it challenging to return results on incidental and negative findings, or to unaffected individuals. Furthermore, about a quarter did not recognize the importance of confirming genetic research results in a clinically accredited diagnostic laboratory prior to disclosure, and critically, access to such laboratories remains low in LMIC. There were also significant knowledge gaps among the respondents regarding their own local legal framework and ethical policy on RoR. Notably, only half ascertained their participants' preferences on RoR during recruitment. These findings represent important gaps in RoR feasibility and readiness within the GP2 community. Based on these observations, we have formulated several recommendations for key next steps to improve RoR workflow in PD genetic research, starting from improvements to the informed consent process, to follow-up planning for RoR, summarized in Table 2. Notably, there remains a wide disparity between LMIC and HIC regarding access to genetic testing and funding; strategies to address these disparities have been previously discussed elsewhere.^22^ While the GP2 is a genetic discovery initiative and not an effort primarily aiming to return genetic results to individual patients, we have also begun to navigate and support RoR by partnering with PDGENEration,^40^ an initiative designed to carefully return genetic results to patients.

**Table 2:** Suggested Next Steps to Improve the ROR Workflow in Parkinson Genetic Research

Improved informed consent processes	This is particularly so for new centers with prospective cohort collection. Ethical documents should ideally have clear statements on ROR practices, including the scope of findings to be returned, and should be compliant with local laws and regulations. Where possible, research participants should be given opportunities to indicate their preferences, including the option of choosing only certain types of findings (e.g., those considered “clinically relevant” to diagnosis, prognosis, and family planning, and/or those considered “actionable” where prevention or treatment is available) to be returned, rather than the conventional “all or none” approach. It is also prudent to consider separate consent forms with clear and appropriate wordings for affected and unaffected research participants
Increased capacity for genetic counselling	This could include the creation of certified training programs, or less formalized in-person or online training courses. The development of genetic counselling toolkits for common genetic abnormalities in PD and PD-related disorders could be helpful. Specific training resources should also be developed for counselling of clinically unaffected individuals. It is important that these training programs and resources should be tailored to cater to the differences in language and culture, and knowledge levels of clinicians and patients, especially in LMIC. The establishment of regional centers and networks for genetic counselling, for example, through collaborations via research consortia (e.g., PD Generation), is a good training and support model, especially for new centers. The use of innovative technology in developing telegenetic consultations may help to increase access to genetic counselling and overcome geographical barriers
Increased capacity to confirm results in a clinically accredited laboratory	A cost-effective approach could involve regional collaboration to establish laboratories with local/regional certifications (or subsidized CLIA certification), thereby making the return of certified genetic results more feasible especially across lower-income regions. Research funding bodies for genetics research should consider funding the ROR processes, especially in LMIC, where access to genetic result validation is low. In some research initiatives (e.g., the PD GENEration [PD GENE] study^40^ and the Rostock International PD study [ROPAD]^13^), genetic testing is performed, at the outset, in accredited laboratories, and at scale. These steps are also crucial in bolstering recruitment for genetics-informed clinical trials of disease-modifying therapies in these underrepresented populations
Improved logistical infrastructure for “recontacting” and ROR processes	Researchers should be encouraged to develop a ROR plan as part of their research study design. Ideally, ethics approval should include provisions for the participants to be recontacted for repeat biological sampling for validation studies as well as participation in further related research (e.g., biomarker studies or clinical trials). Researchers should consider planning a clear pathway for the disclosure of validated genetic results (including who should do this, and when and how to return the results)
Continuous efforts in educating health care professionals and the public about the role of genetics in Parkinson disease	This will foster a more informed and receptive environment for participation in genetics research and in the return of genetic research results

Online surveys offer many advantages including the opportunity to access a large sample of individuals worldwide, automation and consistency in the invitation language, cost and time efficiencies, and convenience for the respondents. By using a personalized email invitation and timely reminders, the response rate to this survey (almost 40%) was higher compared with others in the field (11%–16%).^38^ Crucially, the survey cohort was representative of the professionals involved in PD genetics research, with participation from 147 institutions across 60 countries and 6 continents. Limitations of the survey include the sampling method (i.e., limited to only GP2 members), response bias (e.g., respondents may have more intention to return results compared with nonrespondents), ambiguity when interpreting some questions, and limited depth (responses being based on multiple-choice format). Although this survey mainly targeted GP2 members, the ethical principles, perceptions, and readiness for genetic testing and return of genetic research results are likely to be similar across various PD genetic research programs, therefore, increasing the generalizability of the results from this survey.

In conclusion, this survey highlights the diversity of perceptions, practice, resources, and readiness as well as ethical considerations surrounding the return of genetic research results, among professionals involved in PD genetics research worldwide. Recognizing these complexities and offering tailored strategies that address different needs and frameworks can pave the way for a more effective and ethically sound implementation of RoR, thereby advancing both genetics research and the delivery of personalized medicine.

## Appendix 1 Authors

**Table:**

Name	Location	Contribution
Ai Huey Tan	Division of Neurology, Department of Medicine, Faculty of Medicine, University of Malaya, Kuala Lumpur, Malaysia	Drafting/revision of the manuscript for content, including medical writing for content; major role in the acquisition of data; study concept or design; analysis or interpretation of data
Paula Saffie-Awad	Programa de Pós-Graduação em Ciências Médicas da Universidade Federal do Rio Grande do Sul, Clínica Santa María, Santiago, Chile	Drafting/revision of the manuscript for content, including medical writing for content; study concept or design; analysis or interpretation of data
Artur F Schumacher Schuh	Departamento de Farmacologia, Universidade Federal do Rio Grande do Sul; Serviço de Neurologia, Hospital de Clínicas de Porto Alegre, Porto Alegre, Brazil	Drafting/revision of the manuscript for content, including medical writing for content; study concept or design; analysis or interpretation of data
Shen-Yang Lim	Division of Neurology, Department of Medicine, Faculty of Medicine, University of Malaya, Kuala Lumpur, Malaysia	Drafting/revision of the manuscript for content, including medical writing for content; study concept or design; analysis or interpretation of data
Harutyun Madoev	Institute of Neurogenetics, University of Lübeck, Germany	Major role in the acquisition of data
Azlina Ahmad-Annuar	Department of Biomedical Science, Faculty of Medicine, University of Malaya, Kuala Lumpur, Malaysia	Major role in the acquisition of data; study concept or design
J. Solle	The Michael J. Fox Foundation for Parkinson's Research, New York	Drafting/revision of the manuscript for content, including medical writing for content; major role in the acquisition of data; study concept or design
Claire E. Wegel	Department of Medical and Molecular Genetics, Indiana University, Indianapolis	Major role in the acquisition of data
Maria Leila Doquenia	Institute of Neurogenetics, University of Lübeck, Germany; Department of Neuroscience and Brain Health, Metropolitan Medical Center, Manila, Philippines	Drafting/revision of the manuscript for content, including medical writing for content; major role in the acquisition of data
Sumit Dey	Centre for Preventive Neurology, Wolfson Institute of Population Health, Queen Mary University of London, United Kingdom	Drafting/revision of the manuscript for content, including medical writing for content; analysis or interpretation of data
Maria Teresa Perinan	Centre for Preventive Neurology, Wolfson Institute of Population Health, Queen Mary University of London, United Kingdom; Unidad de Trastornos del Movimiento, Servicio de Neurología y Neurofisiología Clínica, Instituto de Biomedicina de Sevilla, Hospital Universitario Virgen del Rocío/CSIC/Universidad de Sevilla, Spain	Drafting/revision of the manuscript for content, including medical writing for content; analysis or interpretation of data
Mary B. Makarious	Laboratory of Neurogenetics, National Institute on Aging, National Institutes of Health, Bethesda, MD; Department of Clinical and Movement Neurosciences, UCL Queen Square Institute of Neurology, University College London, United Kingdom	Drafting/revision of the manuscript for content, including medical writing for content; analysis or interpretation of data
Brian Fiske	The Michael J. Fox Foundation for Parkinson's Research, New York	Drafting/revision of the manuscript for content, including medical writing for content; analysis or interpretation of data
Huw R. Morris	Department of Clinical and Movement Neurosciences, UCL Queen Square Institute of Neurology, University College London, United Kingdom	Drafting/revision of the manuscript for content, including medical writing for content; analysis or interpretation of data
Alastair J. Noyce	Centre for Preventive Neurology, Wolfson Institute of Population Health, Queen Mary University of London, United Kingdom	Drafting/revision of the manuscript for content, including medical writing for content; analysis or interpretation of data
Roy N. Alcalay	Department of Neurology, Columbia University Irving Medical Center, New York; Movement Disorders Division, Neurological Institute, Tel Aviv Sourasky Medical Center and Tel Aviv School of Medicine, Tel Aviv University, Israel	Drafting/revision of the manuscript for content, including medical writing for content; study concept or design; analysis or interpretation of data
Kishore Raj Kumar	Molecular Medicine Laboratory and Neurology Department, Concord Clinical School, Concord Repatriation General Hospital, The University of Sydney; Translational Neurogenomics Group, Genomic and Inherited Disease Program, Garvan Institute of Medical Research, Darlinghurst; St Vincent’s Healthcare Campus, Faculty of Medicine, UNSW Sydney, Darlinghurst, New South Wales, Australia	Drafting/revision of the manuscript for content, including medical writing for content; major role in the acquisition of data; study concept or design; analysis or interpretation of data
Christine Klein	Institute of Neurogenetics, University of Lübeck, Germany	Drafting/revision of the manuscript for content, including medical writing for content; study concept or design; analysis or interpretation of data

## Appendix 2 Coinvestigators

**Table:**

Coinvestigators are listed at Neurology.org/NG.

## Glossary

HIC: high-income countries
LMIC: low- and middle-income countries
PD: Parkinson disease
RoR: return of results
